# Prurigo in Children of Tropical Zone: Epidemiological, Clinical, and Etiological Characteristics in a Dermatology Department in Cotonou (Benin)

**DOI:** 10.1155/2019/2673981

**Published:** 2019-10-31

**Authors:** Bérénice Dégboé, Christiane Koudoukpo, Nina Maffo, Serge Otémé, Alida Kouassi, Fabrice Akpadjan, Nadège Agbéssi, Nadège Elégbédé-Adégbitè, Jojo Kalogama, Odile Houngbo, Hugues Adégbidi, Félix Atadokpèdé

**Affiliations:** ^1^Department of Dermatology-Venereology of the National and Teaching Hospital HKM, Cotonou, Benin; ^2^Faculty of Health Sciences, University of Abomey-Calavi, Cotonou, Benin; ^3^Department of Dermatology-Venereology of the Departmental and Teaching Hospital of Borgou-Alibori, Cotonou, Benin; ^4^Faculty of Health Sciences, University of Parakou, Parakou, Benin

## Abstract

**Introduction:**

The objective of this work is to document the epidemiological, clinical, and etiological features of prurigo in children.

**Methods:**

This is a descriptive and retrospective study done from January 2013 to September 2018 in the Dermatology Department of National and Teaching Hospital HKM of Cotonou. All children from 0–18 years diagnosed clinically with prurigo were the study sample. Visual analog scale was used to assess the severity of pruritus. The data were entered and analyzed with EpiData and Epi Info 7 software.

**Results:**

The prevalence of prurigo was 14.9% (234/1565) in the pediatric population. The mean age of the children at the onset of the disease was 5.4 years ± 4.9 years. Their sex ratio was 0.8. Pruritus was reported in 97.8% of cases; it was moderate in 50% and severe in 50%. Several phenotypes were described, including erosivo-crusted prurigo (36.3%) and papulo-vesicular prurigo (32%). Frequently observed clinical forms were chronic (44.4%), acute (38.9%), impetiginized (8.1%), and lichenified (4.3%). Prurigo predominated on the lower limbs (74.8%), upper limbs (47.9%), and buttocks and trunk (24.8% each). The main etiologies were prurigo strophulus (PS) (55.5%), scabiosis (20.5%), prurigo of Besnier (10.7%), and hookworm cutaneous larva migrans (HCLM) (8.5%). The PS was seasonal (*p*=0.036), while prurigo of Besnier, scabies, and HCLM were perennial.

**Conclusion:**

The main etiologies of prurigo in the study participants were PS, prurigo of Besnier, scabiosis, and HCLM. It affected with predilection the limbs of children of less than 5 years. Prurigo was almost always itchy and often evolved in an acute or chronic mode.

## 1. Introduction

Prurigo is a universally used term characterized by pruritic skin disease and is often papulo-vesicular or excoriated [1, 2]. Despite this broad and all-encompassing definition, it is often referred to in the literature as prurigo strophulus.

It is a frequent and ubiquitous condition with multiple and varied etiologies affecting both sexes (male and female) and all ages [2–4].

The clinical aspect of prurigo varies very little. However, it presents particularly diagnostic problem because the etiologies are multiple and can be entangled. In its chronic form in black adult Africans, it is highly suggestive of human immunodeficiency virus (HIV) infection which was reported as early as 1986 in Haiti and Africa [3–7]. In general, its etiologies are most often different in children and adults, but depends on climatic and environmental conditions [3, 7–9]. Because of its itchy and often chronic nature, complications remain more important in children [3, 4, 10]. The present study aims to describe the epidemiological, clinical, and etiological features of prurigo in children in tropical environments, particularly in a dermatology department in Cotonou.

## 2. Methods

This is a retrospective, descriptive, and analytical study done for 5 years and 9 months (January 2013–September 2018) in the Department of Dermatology-Venerology of the National and Teaching Hospital Hubert Koutoukou Maga (CNHU-HKM) of Cotonou. From the pediatric population aged 0–18 years, all children diagnosed clinically with prurigo were selected. The demographic, clinical, and etiological data of prurigo were collected on a pre-established survey form. Visual analog scale was used to assess the severity of pruritus. The data were entered and analyzed anonymously with EpiData and Epi Info 7 software.

## 3. Results

The prevalence of prurigo was 14.9% (234/1565) in the pediatric population.  Table 1 shows the distribution by age group. Children aged 0–5 years constituted half of the study population. The mean age of onset was 5.4 years ± 4.9 years. Girls were 54.7% giving a sex ratio of 0.8. The delay before consultation was highly variable with a median of 21.3 days ± 49.9 days.

Pruritus was reported in the majority of patients, 229 patients (97.8%); it was moderate in 50% of patients and severe in others (50%). Five patients (2.2%) who had cicatricial prurigo did not report pruritus during the consultation.

Regular deworming was achieved in 168 children, 71.8%. The absence of regular deworming was reported in 66 children (28.2%).

Several clinical forms were described and are listed in Table 1. There were morphological forms, evolutionary forms, and complicated forms reported in 33 patients (24.1%) with impetiginization, lichenification, and eczematization.

The topography of prurigo (Table 2) predominated on the lower and upper limbs, trunk, and buttocks.

**Figure 1:**
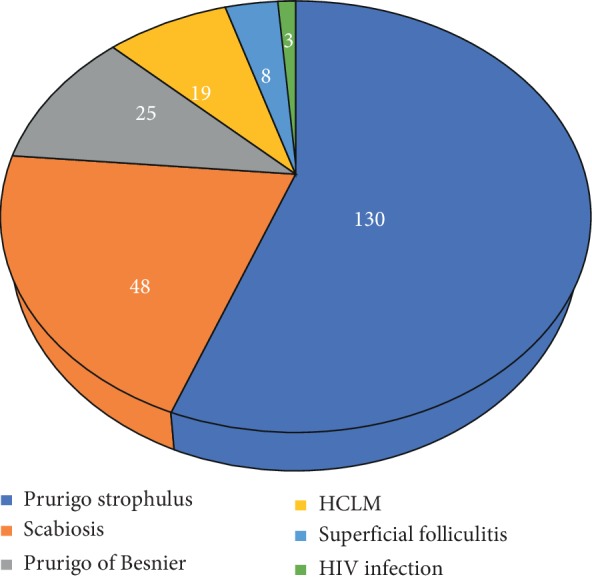
Main etiologies of prurigo in the 234 children with prurigo in the Department of Dermatology of the CNHU-HKM of Cotonou from January 2013 to September 2018.

**Figure 2:**
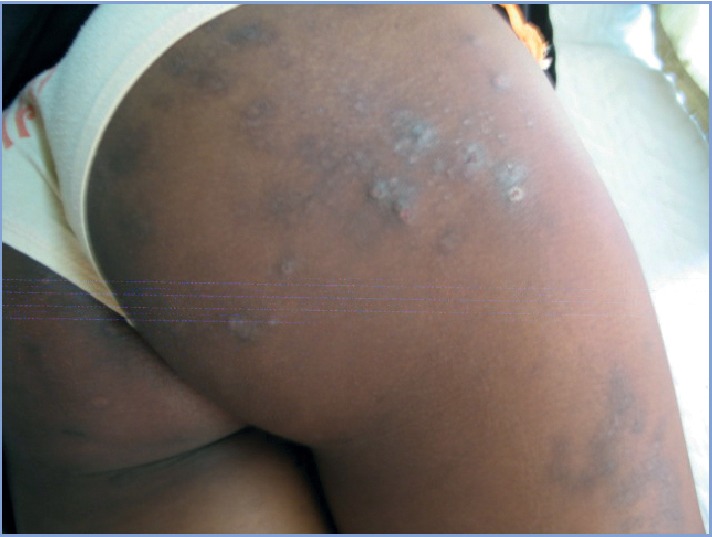
Lesions of prurigo strophulus on the lower limbs in a girl.

**Figure 3:**
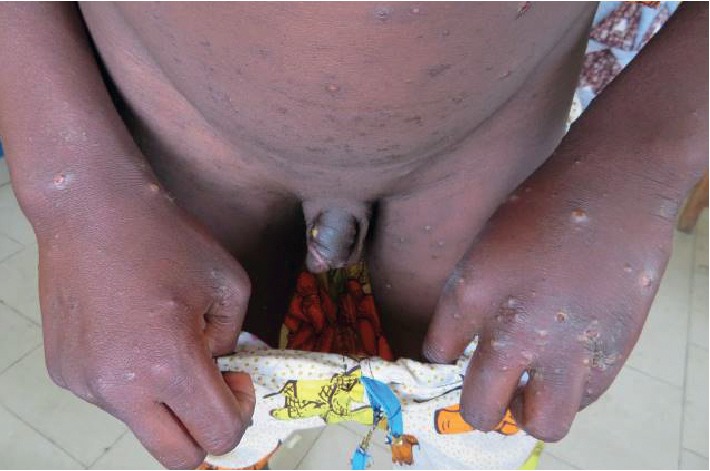
Scabiosis on the upper limbs and external genitals in a boy.

**Figure 4:**
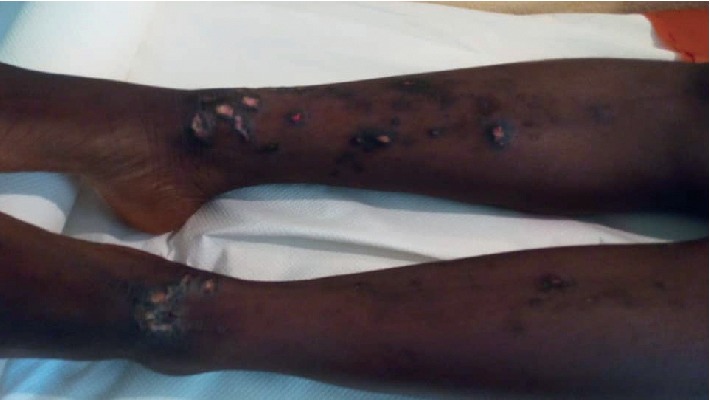
Prurigo of Besnier on the lower limbs in a girl.

**Figure 5:**
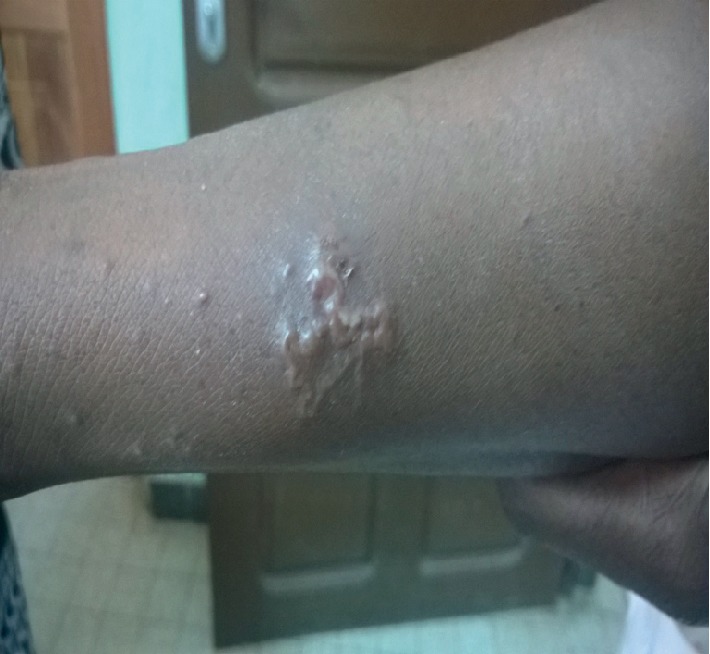
Hookworm cutaneous larva migrans on the upper limb in a boy.

The main etiologies (Figures 1–5) found in our pediatric series were prurigo strophulus (PS) (130; 55.5%), scabiosis (48; 20.5%), prurigo of Besnier (PB) (25; 10.7%), and hookworm cutaneous larva migrans (HCLM) (19; 8.1%). Prurigo-associated HIV infection was reported in 3 children (1.3%).

PS and HCLM significantly increased in children under 5 years (0.03 < *p* < 0.049), whereas for other etiologies (scabies, prurigo of Besnier, and superficial folliculitis), there was no predilection for age range. The different etiologies found affected both sexes without preference, except for HCLM, which was significantly found in boys (*p*=0.01). PS and superficial folliculitis were seasonal, mostly occurring during the dry season (0.004 < *p* < 0.036), while prurigo of Besnier, scabiosis, and HCLM were perennial. In patients with PS (*n* = 130), the predominant atopic antecedents were rhinitis (33.8%), sinusitis (32.3%), and asthma (22.3%). For those with prurigo of Besnier (*n* = 25), rhinitis accounted for 40%, sinusitis, 24%, and asthma, 20%. Atopic antecedent frequencies were much lower for other etiologies.

## 4. Discussion

This study has some weaknesses because of its retrospective nature and absence of allergological or biological tests to confirm etiological diagnoses. However, with the experience of our dermatologists, we have been able to document the epidemiological, clinical, and etiological characteristics of prurigo in children.

The prevalence of prurigo in the pediatric population was 14.9% as previously reported in the service [11].

The mean age of onset (5.4 years ± 4.9 years) found in our study is reported by most authors [2–4, 7, 11, 12]. This observation could be explained on the one hand by the fact that this period corresponds to that of the first sensitizations to environmental allergens, and on the other hand, the immune system of children in this age group is still immature [13].

We have had a predominance of girls as some authors [3, 4, 11, 12], while other authors have reported a higher frequency of boys [2]. This can be explained by the fact that, in the African tropical zone, girls' clothes are often less covering than those of boys. In addition, the appearance of unattractive lesions incites parents to take girls more often than boys for consultation [3, 4, 11, 12].

Pruritus is the best sign of prurigo. It is absent in 5 patients and could be related to the cicatricial stage observed.

Hookworms are common in Africa, especially among children. They may, in some cases, manifest as pruritus, urticaria, or prurigo [14]. The absence of regular deworming in 66 children (28.2%) could be considered responsible or cofactor of prurigo in children.

When examining a patient with prurigo, it is difficult to clearly identify, from the appearance of the lesions, dermatoses defined clinically, histologically, or etiologically. It is also difficult to assert its primitive or secondary character [2, 3, 10, 15]. In our series, erosivo-crusted and papulo-vesicular forms, which were the most frequent morphological forms, could not orient us towards a given etiology. However, the serpiginous papular form, suggestive of a larva migrans, allowed us to retain the diagnosis of HCLM in 19 patients.

The elective topography of the limbs could lead to a prurigo by insect bites, very common in tropical areas in children [3, 4, 7, 12]. However, the involvement of the trunk and buttocks, which are often protected areas, does not exclude a prurigo by insect bites because the topography is a function of the habits of the biting insect [2, 4].

In this event, we mention the hypersensitivities secondary to the bites of house dust mites but also that of scabies. However, other etiologies excluding sting by arthropods can be evoked. The typical papulo-vesicular appearance is observed in one in three patients because patients wait an average of 21.3 days ± 49.9 days to consult.

Pruritus contributes to excoriating lesions, exposing children to complications such as impetiginization [16, 17] in 19 patients (8.1%) and lichenification (10 cases; 4.3%) in the long term. Self-medication, which is a phenomenon frequently reported in our regions, could also induce eczematization [16] and secondarily lichenification [18, 19].

Currently, the pathophysiology of prurigo is not clearly understood. Its chronological character may be an argument in favor of etiologies. Thus, the etiologies of acute prurigo (representing 38.9% of our series) that are reported in previous studies are most often ectoparasitosis such as scabies found in 20.5% of children, insect bites, lice, and fleas or more rarely an external irritant factor such as pollen or dust. [2, 20, 21]. We associate in this particular climatic context the HCLM that was noted in 8.1% of children. In this case, the skin reaction at the puncture site is thought to be related to the combination of an acute and innate reaction to the “toxicity” of the foreign protein, associated with a variable degree of skin reactivity of the patient [22].

Subacute prurigo is recurrent and is more rarely found in children. This has been confirmed in our series because it is found in 15% of children. It occurs preferentially in middle-aged women with atopic sites or comorbidities such as psychiatric disorders, diabetes, and renal or hepatic insufficiency [23]. This entity is often controversial by some authors, who equate it with a chronic prurigo.

The pathophysiology of chronic prurigo in adults according to Schuhknecht et al. would rely on the interaction between chronic inflammation, the secretion of neuromediators, and the neuropathy of the small cutaneous fibers observed in cases of nodular prurigo [24]. In children, we believe that chronic prurigo (44.4% of our series) is secondary to a delayed hypersensitivity reaction associated with the persistence of exposure to environmental allergens. The prurigo strophulus (55.5%) and prurigo of Besnier (20.5%) both observed in 66.3% of children could respond well to this mechanism. Maridet et al. describe this pathophysiology as complex, mixed, and evolutionary. It involves innate and acquired mechanisms of immediate and delayed hypersensitivity that could explain the recurrence of relapses and the progression towards chronicity [2, 21, 25]. A natural improvement of the attacks is possible by desensitization which can take months or even years [2].

Atopic terrain would be an important cofactor of these hypersensitivities as shown by our results, where atopic pathologies were associated with prurigo strophulus and prurigo of Besnier between 20 and 40% of cases [2, 4, 11, 12, 18, 21].

As reported in the literature, PS (55.6%) was the main etiology of prurigo in children [2, 3, 25, 26]. Its characteristic lesion is the sero-papular of Tommasoli [3, 4]. It is most often recognized as seasonal, either in the hot season [2, 4, 21] or in the rainy season [12], which makes it an environmental dermatosis. In our series, the majority of children with PS started their thrust during the hot season (*p*=0.036). The rainy season would be a breeding season and the season for the growth of most arthropods, while the dry season is the period during which the insect population peaks, allowing them to adapt to changing conditions like severe climatic conditions [21]. In addition, during the hot season, children often wear less covering clothing, which increases insect bites during this period.

Scabiosis observed in 20.5% of children is a highly contagious ectoparasitosis that is quite common in children [16, 27, 28]. It constitutes with HCLM the two frequent ectoparasitosis, source of lesions of prurigo in children [16, 27–29]. Promiscuity and poor hygiene are the contributing factors that are constantly reported [3, 16, 17, 27, 28].

Prurigo of Besnier or prurigo diathesis described by Besnier in 1892 is a clinical form of atopic dermatitis characterized by the presence of prurigo lesions and lichenification [30]. However, it is not yet fully studied in Black Africa. Lesions often extend beyond exposed areas [2, 18, 30–32]. The perennial character of prurigo of Besnier is due to the permanent presence of allergens, especially pneumallergens (pollen and mites) in the environment; hence, it is associated with aeroallergen sensitization diseases [2, 18] such as sinusitis, allergic rhinitis, and asthma in this study. This study confirms that prurigo associated with HIV infection is rare in children, unlike adults in sub-Saharan regions [3, 4].

## 5. Conclusion

In our tropical African context in Benin, prurigo most commonly affects children under 5 years, on the limbs, trunk, and buttocks. The main etiologies were prurigo strophulus, atopic dermatitis, scabiosis, and hookworm cutaneous larva migrans. These pathologies are mainly due to immunoallergic and infectious mechanisms. The evolution was most often in an acute mode for ectoparasitoses like scabies and HCLM and chronic mode delayed hypersensitivity during prurigo strophulus and prurigo of Besnier.

## Figures and Tables

**Table 1:** Distribution according to the age groups and the different clinical forms of prurigo among the 234 children in the Department of Dermatology of the CNHU-HKM of Cotonou from January 2013 to September 2018.

	Size	Proportion (%)
Age (years)		
0–5	117	50
6–10	58	24.8
11–13	22	9.4
14–18	37	15.8

Gender		
Male	106	45.3
Female	128	54.7

Clinical forms		
Erosivo-crusted	85	36.3
Papulo-vesicular	75	32
Morphological forms (234; 100%)		
Macular	35	15
Serpiginous papular	19	8.1
Squamous-crusted	10	4.3
Pustular	10	4.3
Evolutionary forms (234; 100%)		
Chronic	104	44.4
Acute	89	38
Subacute	35	15
Cicatricial	6	2.6
Impetiginization	19	8.1
Complicated forms (33; 24.1%)		
Lichenified	10	4.3
Eczematization	4	1.7

**Table 2:** Distribution according to the topography of prurigo in the 234 children in the Department of Dermatology in CNHU-HKM of Cotonou from January 2013 to September 2018.

	Size	Proportion (%)
Legs	175	74.8
Forearm	112	47.8
Arms	101	43.2
Feet	98	41.8
Hands	77	32.9
Trunk	58	24.8
Buttocks	58	24.8
Thighs	58	24.8
Face	24	10.3
External genitals	24	10.3
Neck	12	5.1
Scalp	7	3

## Data Availability

No data were used to support this study.
